# Fuzzy rank-based fusion of CNN models using Gompertz function for screening COVID-19 CT-scans

**DOI:** 10.1038/s41598-021-93658-y

**Published:** 2021-07-08

**Authors:** Rohit Kundu, Hritam Basak, Pawan Kumar Singh, Ali Ahmadian, Massimiliano Ferrara, Ram Sarkar

**Affiliations:** 1 Department of Electrical Engineering, Jadavpur University, Kolkata, 700032 India; 2 Department of Information Technology, Jadavpur University, Kolkata, 700106 India; 3 Institute of IR 4.0, The National University of Malaysia (UKM), 43600 Bangi, Selangor Malaysia; 4 Department of Law, Economics and Human Sciences & Decisions Lab, Mediterranea University of Reggio Calabria, 89125 Reggio Calabria, Italy; 5 Department of Computer Science and Engineering, Jadavpur University, Kolkata, 700032 India

**Keywords:** COVID-19, Deep learning, Convolution neural networks, Ensemble, Gompertz function, Computer science, Scientific data, Statistics, Diseases, Medical research

## Abstract

COVID-19 has crippled the world’s healthcare systems, setting back the economy and taking the lives of several people. Although potential vaccines are being tested and supplied around the world, it will take a long time to reach every human being, more so with new variants of the virus emerging, enforcing a lockdown-like situation on parts of the world. Thus, there is a dire need for early and accurate detection of COVID-19 to prevent the spread of the disease, even more. The current gold-standard RT-PCR test is only 71% sensitive and is a laborious test to perform, leading to the incapability of conducting the population-wide screening. To this end, in this paper, we propose an automated COVID-19 detection system that uses CT-scan images of the lungs for classifying the same into COVID and Non-COVID cases. The proposed method applies an ensemble strategy that generates fuzzy ranks of the base classification models using the Gompertz function and fuses the decision scores of the base models adaptively to make the final predictions on the test cases. Three transfer learning-based convolutional neural network models are used, namely VGG-11, Wide ResNet-50-2, and Inception v3, to generate the decision scores to be fused by the proposed ensemble model. The framework has been evaluated on two publicly available chest CT scan datasets achieving state-of-the-art performance, justifying the reliability of the model. The relevant source codes related to the present work is available in: GitHub.

## Introduction

COVID-19 is considered one of the most infectious diseases of the 21st century that has brought the entire human society to a standstill. The spread of the novel coronavirus that started in Wuhan, China in December 2019, has already caused 87 million infected cases and nearly 2 million fatalities worldwide, as of January 2021. The epidemic has already caused severe damage to the human economy all over the world and the health system has been devastated due to the shortage of intensive care units (ICUs). The main concern in this is the uncontrolled, undetected spread of the virus.

The existing tests for the detection of COVID-19 consist of mainly swab-based Reverse Transcription Polymerase Chain Reaction (RT-PCR) test^1^, and blood sample-based antibody test^2^. The RT-PCR test takes a long time to produce the results, causing a delay in prognosis and diagnosis of the patients and assessment of the severity of the disease. Besides, in the case of over-populated, developing countries RT-PCR tests cannot be conducted on a large scale due to the shortage of apparatus. Therefore, there is a need for some alternative methods for the detection of COVID-19.

**Figure 1:**
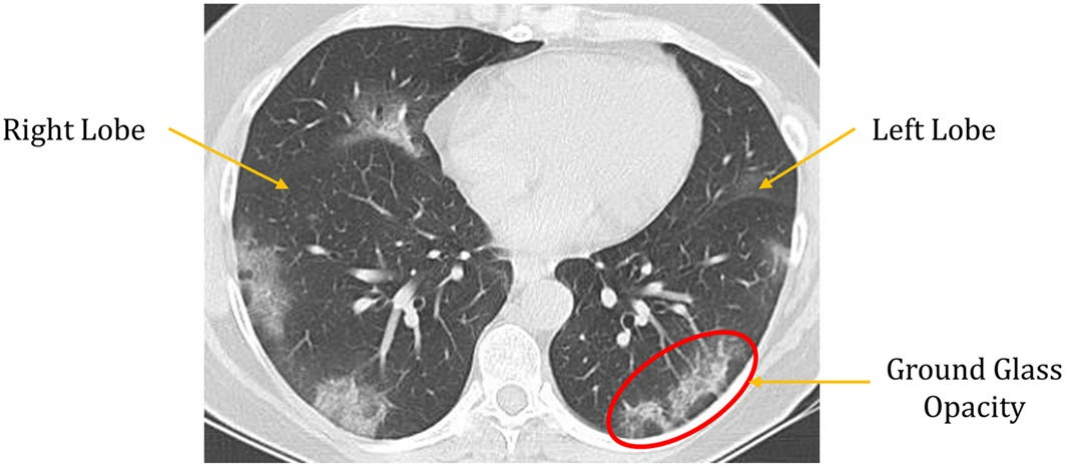
Example of a COVID infected Lung CT-scan image. The CT-scan image has been taken from the SARS-COV-2 dataset^15^. The ground glass opacity marked with the red circle in the image is the distinguishing feature of the COVID-19 infection.

Examination of chest X-Ray or CT scan images can be one such alternative^3,4^. This method is much faster and easily accessible for patients from different economical backgrounds. An example of a lung CT-scan image of a COVID-19 infected patient is shown in Fig. 1. However, instead of expert radiologists or physicians, another way to determine the infection is by using Artificial Intelligence, which augments the physicians’ efforts, and it has been proven to be an effective alternative in other biomedical applications. Data mining or Machine Learning is a useful tool that has an advantage over traditional methods to extract features (like Gabor features, Gray-Level Co-occurrence matrix features, etc.) from medical images^5–7^. It is also practicable to analyze medical image datasets in hospitals with huge volume and variations. There are different data mining methods like KNN and ANN-based classifiers^8,9^, Support Vector Machine (SVM)^10,11^, Bayesian method^12^, decision tree, etc. that have already been used in COVID-19 detection task. Ensembling decision scores from different Transfer Learning-based CNN base models have been practised widely in recent years^13^. However, in this paper we propose screening of Covid-19 CT scans by utilizing a less explored strategy: by generating fuzzy ranks using the Gompertz function^14^, a mathematical model which has been not been explored previously in this domain, adding to the novelty of this research.

Ensemble learning is used to fuse the salient properties of its constituent models and enhances the overall performance, making better predictions than the individual contributing models. They are robust in the sense that ensembling reduces the spread or dispersion of the individual models’ predictions. Ensemble models achieve superior performance by minimizing the variance of prediction errors by adding some bias to the competing base learners. In this research, we have introduced a Fuzzy ranking method using the Gompertz function. The advantage of such fusion is that it uses adaptive weights based on the confidence scores of each classifier used to form the ensemble in order to generate the final prediction of each sample. The Gompertz function^16^ was originally formulated to map a collection of data in life tables through a single function, and was proposed based on the assumption that mortality decreases exponentially as a person ages, and then saturates to an asymptote. Such a function can be useful for fusing the confidence scores of classifiers for a complex image classification problem where the confidence score for a prediction class by a classifier hardly becomes truly zero, but some small value.

CT^17,18^ imaging are proven to have more discriminating patterns to ensure more sensitivity and specificity^19–21^ as compared to the traditional RT-PCR method^22^. Therefore Artificial Intelligence (AI) has been widely used to extract patterns from the imaging datasets available to complement and augment the early detection of COVID-19. In literature, there exist numerous applications of Machine Learning^10,23,24^ and Deep Learning^25–27^.

Jaiswal et al.^28^ and Das et al.^29^ used transfer learning with DenseNet-201 for COVID classification on the SARS-COV-2 CT-scan dataset. Panwar et al.^30^ used a pre-trained VGG-19 network and added five more fully connected layers to the original structure to classify COVID CT samples. Karbhari et al.^31^ proposed Auxiliary Classifier GAN (ACGAN) to generate synthesized chest radiograph images to mitigate the problem of scarcity of available data and uses a classifier to perform classification on the synthesized data. Angelov et al.^32^ used the GoogLeNet architecture to extract deep features, however, they trained the model from scratch rather than loading the pretrained ImageNet weights. The deep features extracted were used to train an MLP classifier^33^ for the final classification on the chest CT-images dataset.

### Motivation and contributions

The COVID-19 global pandemic forced the medical workers to devote their time attending to patients with risk to their own lives, to not only COVID patients but also to attend to other disease infected people. Although extensive research is being carried out to develop a vaccine, it will take a long time to reach every citizen, and thus the need for the spread of the coronavirus is still of prime importance especially with new strains of the virus emerging across the world. The RT-PCR testing process is tedious and time-consuming and is only 71% sensitive to COVID-19. Keeping these facts in mind, in this paper, we develop a framework for the classification of COVID-19 patients from Non-COVID patients based on chest CT-scan images. The ensemble framework proposed can be used as a plug-and-play model by saving the model weights and passing the test images through the framework to generate the predictions. This allows the proposed framework to be readily used by non-experts to generate predictions on new images, making it fit for use in the field. Thus, it is fit for use in the practical field for the Computer-Aided Diagnosis of COVID-19.

Highlights of the proposed work are as follows:

1. For end-to-end classification using a deep learning model, a large amount of data is required, which is often not available in the biomedical domain, so we resort to using transfer learning to generate the initial decision scores using three standard CNN models: VGG-11, Wide ResNet-50-2 and Inception v3.
2. A novel ensemble technique has been used to fuse the decision scores of the said models since ensembling is a powerful tool for incorporating the discriminating properties of all the contributing models.
3. The ensemble technique assigns fuzzy ranks to the constituent classifiers employing a re-parameterized Gompertz function. Fuzzy fusion has the advantage of using adaptive priority based on the confidence scores of the classifiers for each sample to be predicted, and hence performs better than traditional ensemble methods.
4. The Gompertz function has exponential growth and then it saturates to an asymptote, which is useful for ensembling the decision scores of the CNN models since the decision score of a class predicted by a classifier rarely becomes truly zero.
5. To evaluate the performance of this framework, two publicly available datasets of chest CT-scan images have been used which are both more widely available test to perform, and also more sensitive to COVID-19. The obtained results outperform the existing methods by a significant margin.

**Figure 2:**
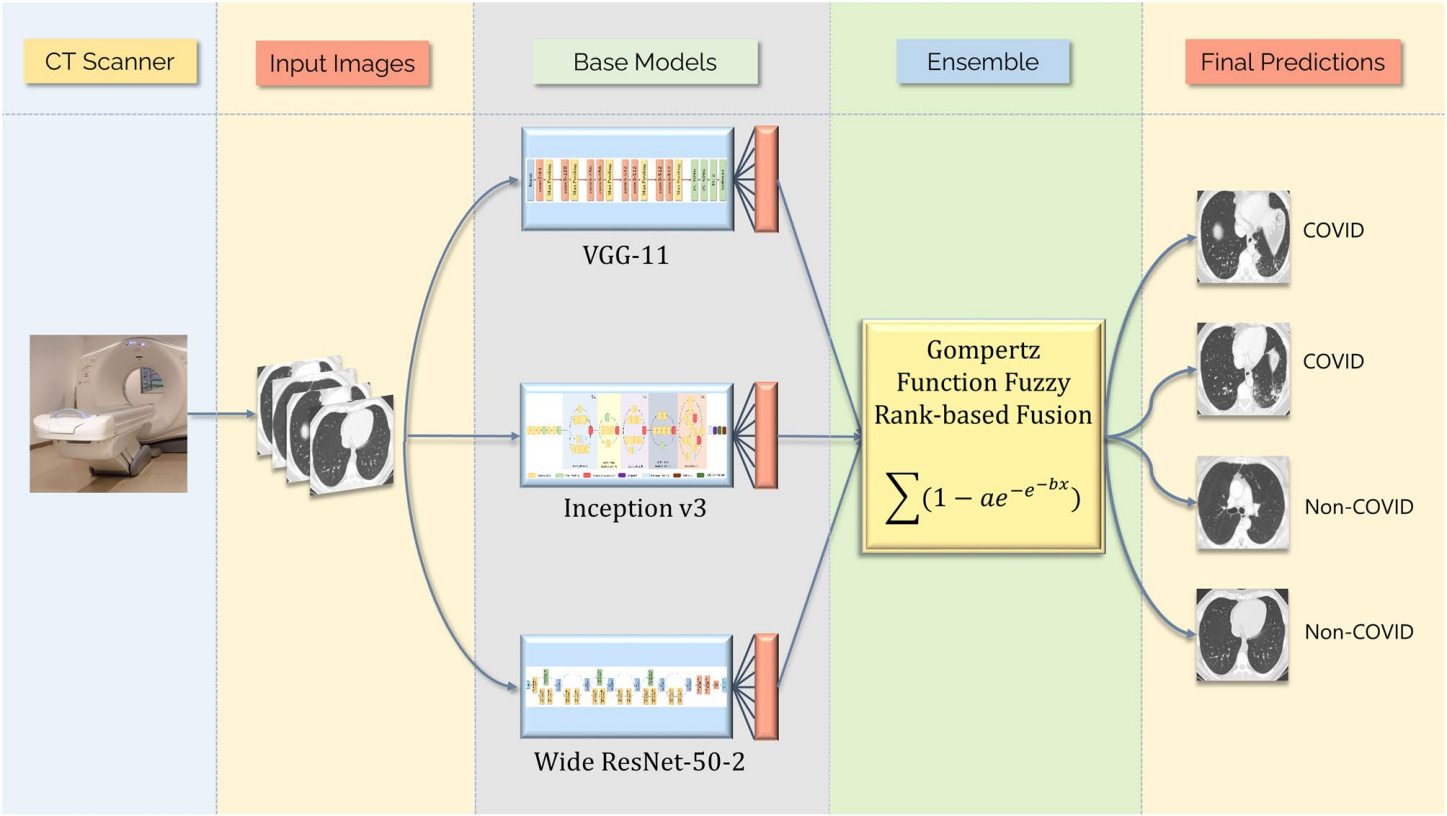
Overall workflow of the proposed framework. The CT Scanner image (open access) is obtained from the Progressive Diagnostic Imaging website^34^ and the chest CT scan images are from the SARS-COV-2 dataset^15^ used in this research.

The overall workflow of the proposed method is shown in Fig. 2.

## Results

### Datasets

To evaluate the performance of the proposed framework, we have used two publicly available datasets, namely the SARS-COV-2 dataset by Soares et al.^15^ and the Harvard Dataverse chest CT dataset^35^. Both datasets have unequal distribution of images, as seen in Table 1. The Harvard Dataverse dataset has been posed as a 2-class problem with COVID and Non-COVID classes for this study.

**Table 1:** Distribution of images in the two datasets used in the present work.

Dataset	Category	Total no. of images	No. of images in Train set	No. of images in Test set
SARS-COV-2	COVID	1252	876	376
	Non-COVID	1229	860	369
Harvard Dataverse	COVID	2167	1517	650
	Non-COVID	2005	1404	601

### Implementation

In the present research, the VGG-11 model has been employed instead of the other deeper CNN variants like VGG-13, VGG-16 or VGG-19 since the performance increment by the deeper models are only nominal while being more computationally expensive, as can be seen from Table 2. We can notice from Table 2 that even though VGG-13 and VGG-16 have about 1M and 6M more parameters than the VGG-11 variant, the increase in accuracy is nominal (only 0.13% for VGG-13 and 0.4% for VGG-16). On the other hand, the VGG-19 model having 11 million more parameters than the VGG-11 model has dropped in performance. Since the amount of data available is low in the medical domain, only linearly increasing the number of layers does not make the model more capable of capturing the complex data pattern. Based on these experimental results, along with the VGG-11 model, we ensemble the other two said CNN models to capture the complementary information from the data.

**Table 2:** Performance (measured in terms of accuracy) provided by the different VGG variants along with their number of parameters on the SARS-COV-2 dataset.

Model	Accuracy (%)	Number of Parameters (in millions)
VGG-11	96.38	132.86
VGG-13	96.51	133.05
VGG-16	96.78	138.42
VGG-19	95.17	143.67

**Table 3:** Results obtained by the ensemble of WideResNet-50-2 and Inception v3 with varying VGG models on the SARS-COV-2 dataset.

VGG Model Used	Accuracy (%)	Precision (%)	Recall (%)	F1-Score (%)
VGG-11	98.93	98.93	98.93	98.93
VGG-13	98.25	98.26	98.25	98.25
VGG-16	98.12	98.13	98.12	98.12
VGG-19	97.04	97.05	94.04	97.04

To further justify the choice of VGG-11 among its other popular variants, we perform the proposed ensemble method using WideResNet-50-2 and Inception v3 models with the different VGG models. The results of the ensemble are shown in Table 3. From the table, we can see that performing the ensemble with VGG-11 gives the best results, indicating that complementary information is obtained by the VGG-11 model with respect to WideResNet-50-2 and Inception v3 models, thus enhancing the performance of the individual learners through the ensemble. Hence, in the present work, we have used three CNN models to form the ensemble: VGG-11, WideResNet-50-2 and Inception v3.

**Figure 3:**
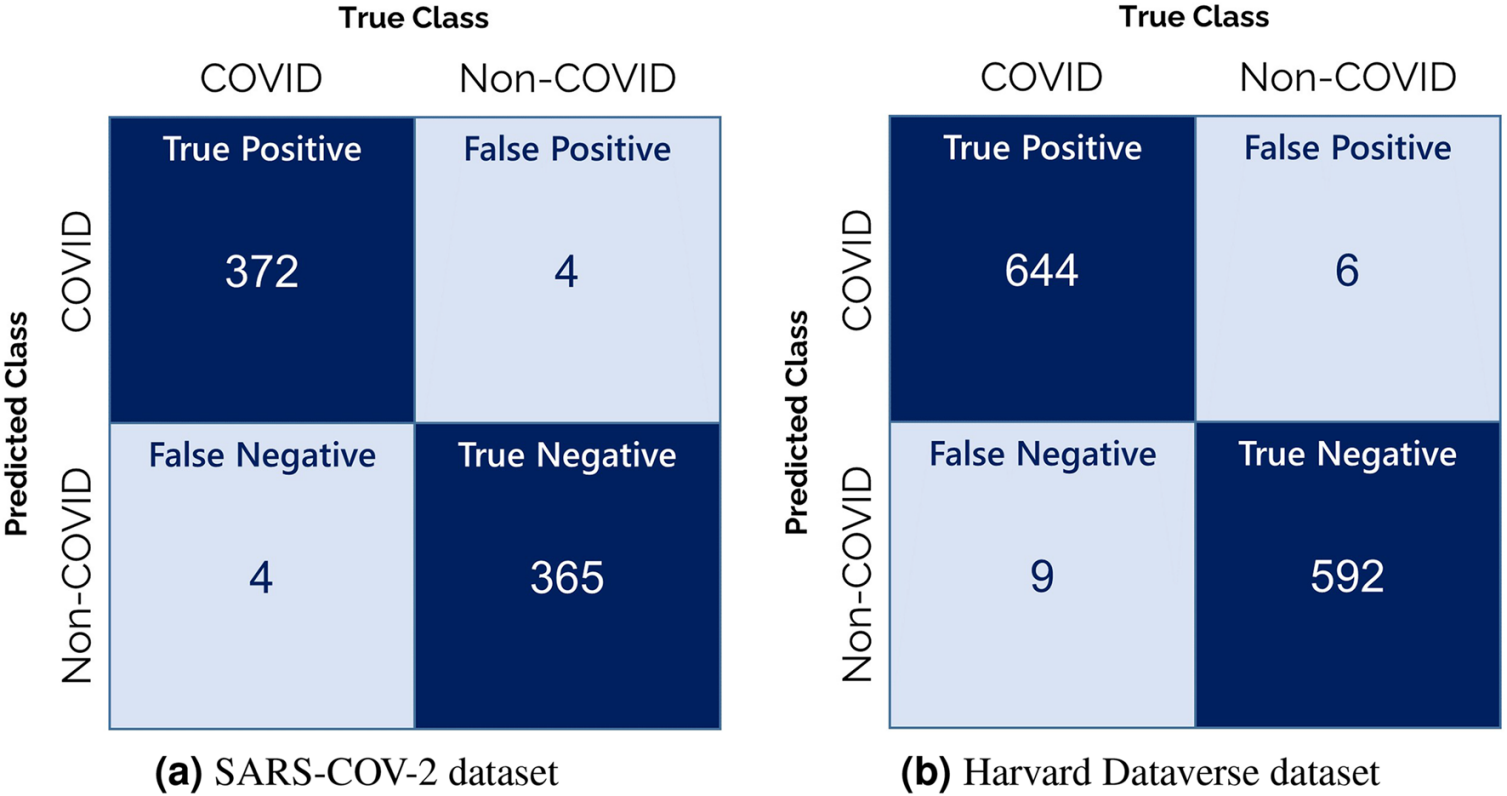
Confusion matrices obtained by the proposed ensemble model on the two datasets considered in the present work.

The results obtained by the proposed ensemble framework on two publicly available datasets are shown in Table 4. Both the class-wise results and the net results are given in the table. High classification accuracies of 98.93% on the SARS-COV-2 dataset and 98.80% on the Harvard Dataverse dataset as well as high sensitivity values of 98.93% and 98.79% respectively on both the datasets have been achieved, thus proving the model to be reliable. The transfer learning-based CNN models used in the framework to generate decision scores have been fine-tuned for 50 epochs each using the Stochastic Gradient Descent optimizer with an initial learning rate of 0.001. Figure 3 shows the confusion matrices obtained on the two datasets used. Although the classification is not perfect, the number of misclassified samples as compared to the correctly classified samples between the “COVID-19” and “Non-COVID” classes are pretty low.

**Table 4:** Results obtained by the proposed ensemble framework on the test sets of both SARS-COV-2 and Harvard Dataverse datasets.

Dataset	Class	Accuracy (%)	Specificity (%)	Precision (%)	Sensitivity (%)	F1 Score (%)
SARS-COV-2	COVID	99.20	98.92	98.68	99.20	98.94
	Non-COVID	98.65	98.94	99.18	98.64	98.91
	Net Results	98.93	98.93	98.93	98.93	98.93
Harvard Dataverse	COVID	99.08	99.00	98.62	99.08	98.85
	Non-COVID	98.50	98.62	99.00	98.50	98.75
	Net Results	98.80	98.82	98.81	98.79	98.80

## Discussion

In this paper, we propose a Fuzzy rank-based fusion of different CNN base models using the Gompertz function, leveraging more extensive features from different CNN modalities. False Positive Rate (FPR) is the phenomenon of classifying a negative quantity as a positive one mistakenly. The high rate of false-positive in medical data analysis can be detrimental, especially in the case of COVID-19 identification, because classifying an infected case as a non-infected one can further spread the disease since the predicted non-infected person will become a super-spreader^36^.

**Figure 4:**
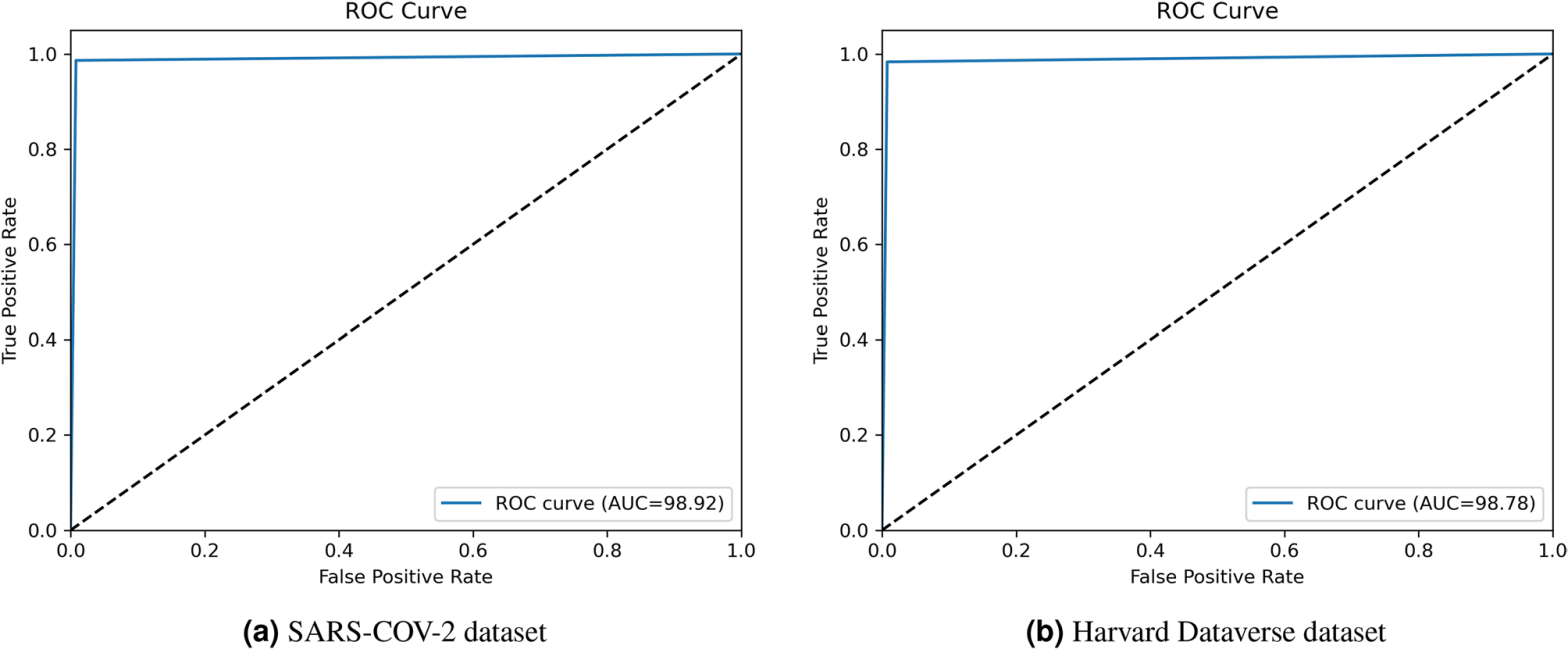
ROC curves obtained by the proposed ensemble model on the two datasets.

The ROC curves obtained on the two datasets using the proposed approach have been shown in Fig. 4. The figures show the False Positive Rate to be significantly low on both the datasets used in this study, manifesting the superiority of the proposed method and its usefulness in medical data analysis. The Area Under the Curve (AUC) values obtained on the corresponding datasets are also mentioned. On the SARS-COV-2 dataset, an AUC value of 98.92% is obtained while on the Harvard Dataverse dataset, the AUC value obtained is 98.78%. In a ROC curve, the higher the value on the X-axis indicates the higher number of false-positive instances than the number of true negative instances. On the other hand, a higher value on the Y-axis suggests a higher number of instances of true positive cases than false negatives. The more the ROC curve is shifted toward the top-left corner of the Cartesian plane, the better is the ability of the classifier to distinguish between positive and negative class samples, because the point (0, 1) on the ROC graph indicates the point of highest sensitivity and specificity. As can be seen from the figure, the ROC obtained on the SARS-COV-2 dataset in Fig. 4a, lies more toward the top-left corner than the ROC obtained on the Harvard Dataverse dataset in Fig. 4b. Thus, we can say that the classifier can distinguish samples better for the SARS-COV-2 dataset and the ROC of it is thus higher than in the case of the Harvard Dataverse dataset.

High sensitivity values obtained by the proposed method on the datasets, as seen from Table 4, indicate robust performance by the ensemble strategy, which even outperforms the RT-PCR testing procedure which has a sensitivity of only 71%. The high accuracies justify the reliability of the framework.

**Figure 5:**
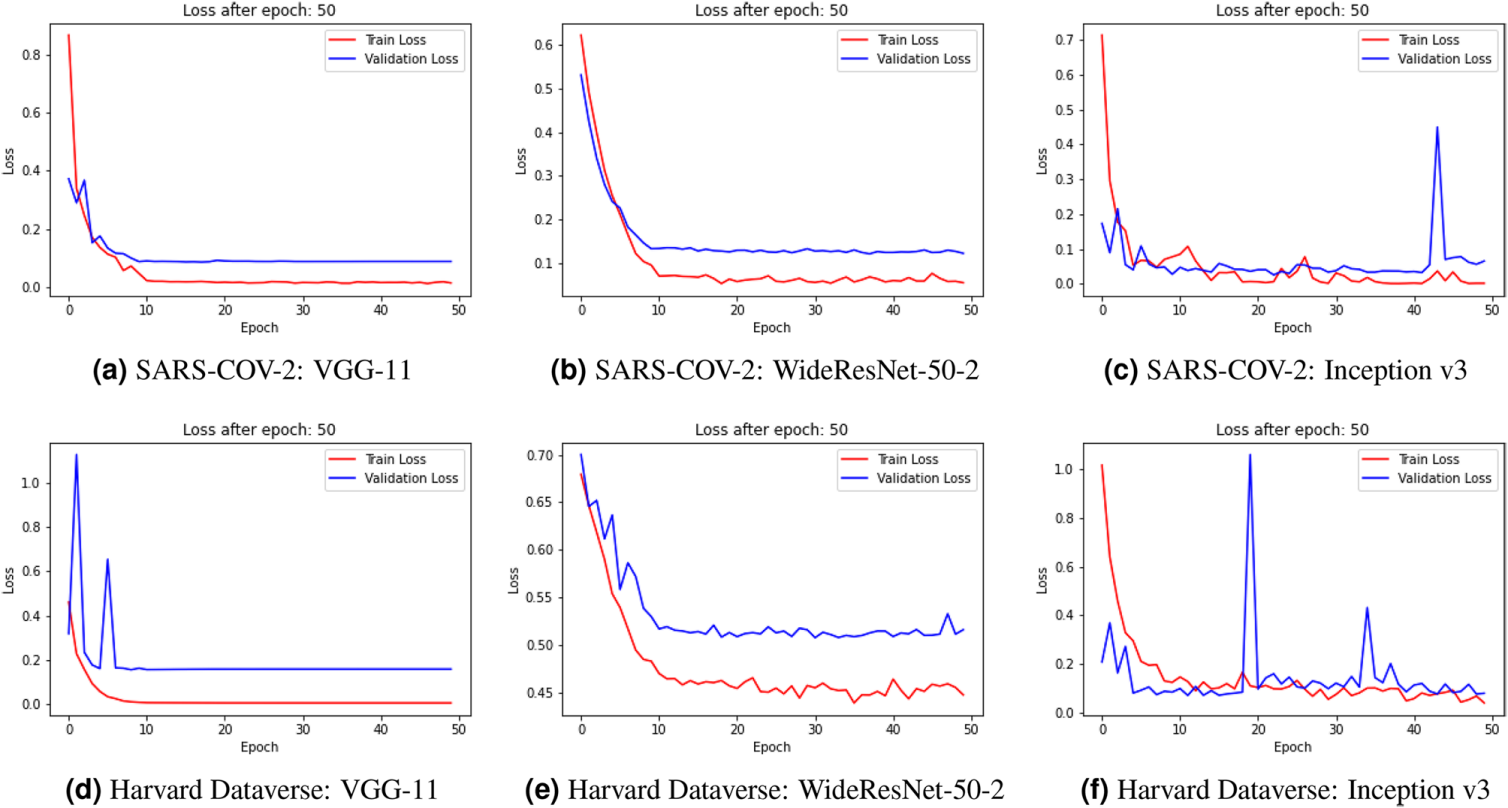
Loss curves obtained on the two datasets used in this research by the base learners used to form the ensemble. (**a**)–(**c**) shows the loss curves on the SARS-COV-2 dataset and (**d**)–(**f**) shows the loss curves on the Harvard Dataverse dataset.

The loss curves obtained by the base learners on the SARS-COV-2 and the Harvard Dataverse dataset used in this research are shown in Fig. 5. Since we use transfer learning models pretrained on the ImageNet dataset, the models only need to be fine-tuned on the COVID dataset. For this, we train the models for 50 epochs each. As seen from the figures, the performance of the models saturates at around 20 epochs, since most of the model weights are already optimized through training on ImageNet. For both the datasets, we observe that in VGG-11 and WideResNet-50-2 there is a problem of slight overfitting of the models, while the problem is not as prominent in the case of Inception v3.

### Comparison with standard CNN Backbones

Transfer Learning is a popular approach for problems in the biomedical image classification domain since often large datasets for training CNNs from scratch are scarce. Transfer Learning allows a model trained on a large dataset to be fine-tuned by the small data in the current problem, making feature learning easier. The proposed ensemble-learning based framework is compared to standard CNN Transfer Learning models including the ones used to construct the ensemble, and the results obtained are presented in Table 5. VGG-11^37^, Wide ResNet-50-2^39^ and Inception v3^41^ have been used in the present study for fusing the decision scores, and clearly, the ensemble of these models outperform the individual models, justifying the reliability of the ensemble framework.

**Table 5:** Comparison of the proposed framework with some standard CNN models.

Standard Model	Accuracy (%)	
	SARS-COV-2	Harvard Dataverse
VGG-11^37^	96.38	95.92
DenseNet161^38^	96.91	95.38
Wide ResNet-50-2^39^	96.78	92.57
ResNet34^40^	96.11	96.87
ResNet152^40^	95.17	94.33
Inception v3^41^	92.15	97.64
Proposed method	98.93	98.80

### Comparison with some conventional ensemble approaches

Ensemble models allow the most important characteristics of all its contributing classifiers to be fused, thus performing superior to the individual models. Many popular ensemble techniques have evolved over the years, some of which have been explored in this study to justify the superiority of the proposed ensemble over existing methods. The fuzzy logic-based ensemble performs especially well since for every sample the confidence in the prediction of a classifier is taken into account to assign weights to the predictions for making the final decision on the class of an image. The results obtained using the same three CNN models to form the ensemble are shown in Table 6, where we can observe that the Gompertz function based decision fusion performs significantly better than the others. The Weighted Average based ensemble approach also achieves good results, but the fuzzy integrals based ensembles (Choquet Integral and Sugeno Integral) perform closest to the proposed ensemble technique. The Weighted Average ensemble is a static process where, at the prediction time, there is no scope for dynamically refactoring the weights to the classifiers. However, fuzzy fusion-based techniques can address this problem and gives priority to the confidence scores making it a superior strategy for the ensemble. Although the Choquet and Sugeno integrals-based ensembles use a similar strategy, the proposed Gompertz function-based fuzzy ranking ensemble still outperforms those, justifying the superiority of the method.

**Table 6:** Comparison of popular ensemble techniques with the proposed Gompertz function based ensemble method.

Ensemble technique	Accuracy (%)	
	SARS-COV-2	Harvard dataverse
Multiplication Rule	95.82	98.24
Maximum	96.78	98.47
Majority Voting	97.65	97.54
Average	97.83	97.91
Weighted Average	98.12	98.64
Choquet Integral	98.52	98.48
Sugeno Integral	98.52	98.48
Proposed Gompertz function based ensemble	98.93	98.80

### Comparison with state-of-the-art

Several COVID detection methods have been proposed in the literature, since the outbreak of the pandemic, although a large fraction of them uses chest X-Ray datasets, which are, in general, less sensitive than chest CT-scan images. The results obtained by some of the recent state-of-the-art methods in literature on the SARS-COV-2 and Harvard Dataverse datasets, used in the current study, are compared with our proposed ensemble model in Table 7. Most of the methods rely on Transfer Learning for classification due to the scarcity of publicly available chest CT data, however, end-to-end classification using transfer learning is not sufficient. Ensembling decision scores from multiple CNN models capture the complementary information provided by the models thus enhancing the overall performance. No published works have yet been found on the Harvard Dataverse dataset to the best of our knowledge, and thus, we compare our results to some popular transfer learning CNN models. The high classification accuracy and sensitivity obtained by the proposed method indicates robustness in performance.

**Table 7:** Comparison of the proposed ensemble framework with state-of-the-art methods on both SARS-COV-2 and Harvard Dataverse datasets.

Dataset	Method	Accuracy (%)	Precision (%)	Recall (%)	F1-Score	Specificity (%)
SARS-COV-2	Silva et al.^42^	97.89	95.33	97.60	96.45	–
	Horry et al.^43^	97.40	99.10	95.50	97.30	–
	Halder et al.^44^	97.00	95.00	98.00	97.00	95.00
	Jaiswal et al.^28^	96.25	96.29	96.29	96.29	96.21
	Sen et al.^27^	95.32	95.30	95.30	95.30	–
	Panwar et al.^30^	94.04	95.00	94.00	94.50	95.86
	Soares et al.^32^	88.60	89.70	88.60	89.15	–
	Proposed method	98.93	98.93	98.93	98.93	98.93
Harvard Dataverse	Krishevsky et al.^45^	94.72	95.17	94.72	94.94	95.17
	Szegedy et al.^46^	92.64	92.64	93.54	93.09	92.64
	Sandler et al.^47^	89.68	88.12	89.68	88.89	89.68
	Proposed Method	98.80	98.82	98.81	98.79	98.80

### Statistical analysis: McNemar’s test

We have performed the McNemar’s test^48^ to statistically analyse the performance of the proposed ensemble method, compared to the constituent models whose decision scores have been used to form the ensemble. Table 8 shows the results of McNemar’s test on both SARS-COV-2 and Harvard Dataverse datasets. To reject the null hypothesis, the p-value in McNemar’s test should ideally be below 5%, and according to Table 8, clearly, for every case the *p* value < 0.05. Thus the null hypothesis is rejected for all the cases. This justifies that the proposed ensemble framework captures the complementary information supplied by the contributing classifiers, and makes superior predictions, thus making the overall model dissimilar to any of the contributing models.

**Table 8:** Results of the McNemar’s Test performed on the individual models of the ensemble, on both datasets: Null hypothesis is rejected for all cases.

McNemar’s Test	*p* value	
Compared with	SARS-COV-2	Harvard Dataverse
VGG-11	4.49E-02	9.50E-03
Wide ResNet-50-2	1.05E-04	1.93E-15
Inception v3	2.88E-02	8.40E-03

## Methods

The proposed framework used for the COVID-19 classification from CT-scan images has two main stages: the generation of confidence scores from multiple models, and the fusion of the decision scores using the Gompertz function employing a fuzzy rank-based scheme for making the final predictions. These two stages are explained in the following sections.

### Generation of confidence score

In the proposed framework, at first, three transfer learning-based CNN models, VGG-11, Inception v3, and Wide ResNet-50-2 are utilized to generate the confidence scores on the sample images. Both the datasets are split into a 70%-30% ratio of train and test sets, and the same sets are used for all the models. The Stochastic Gradient Descent (SGD) optimizer, along with Rectified Linear Unit (ReLU) activation functions are used to fine-tune the networks for 50 epochs each on top of ImageNet weights. The three CNN models are described in brief in the following subsections.

#### VGG-11

**Figure 6:**
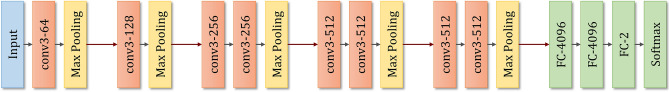
Architecture of the VGG-11 base model.

VGG-11^37^ was proposed for the Visual Recognition Challenge (ILSVRC) in 2014 that was further modified and implemented in several image classification tasks. To exploit the utilization of depth in convolution networks, several other CNN architectures were proposed in the VGG group, we have used the VGG-11 for this purpose, which consists of 8 convolution layers and 3 fully connected (FC) layers, forming 11 layers in total, justifying the nomenclature. The network expects a 3-channel (i.e., RGB image) with dimension $224\times 224$ followed by a series of convolution layers, having a very small receptive field of $3\times 3$ dimension and stride=1, with proper padding. This is followed by non-overlapping Max-pooling layers with size $2\times 2$ and padding size=2 in between some of the convolution layers. The hidden layers of VGG-11 have ReLU activation functions. The architecture of the VGG-11 model is shown in Fig. 6.

#### Wide ResNet-50-2

**Figure 7:**
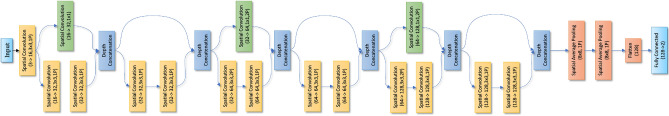
Architecture of the Wide ResNet-50-2 base model.

Wide ResNet architecture was proposed in 2016 by Zagoruyko et al.^39^ 2016. The Wide ResNet model mitigates some of the problems of ResNet^40^ by making the network shallow and wide, thereby reducing the training time and parameters without compromising the performance. The authors of ResNet have made the network shallow to increase the depth, thereby opening up the possibility of the network’s inability to learn anything during training due to the absence of anything to force it to go through the residual block weights. That might lead to a problem of feature reuse: a problem of only a few blocks having important information and the rest of the blocks sharing a small contribution towards the final output. The architecture of the Wide ResNet-50-2 CNN model is shown in Fig. 7.

#### Inception v3

**Figure 8:**
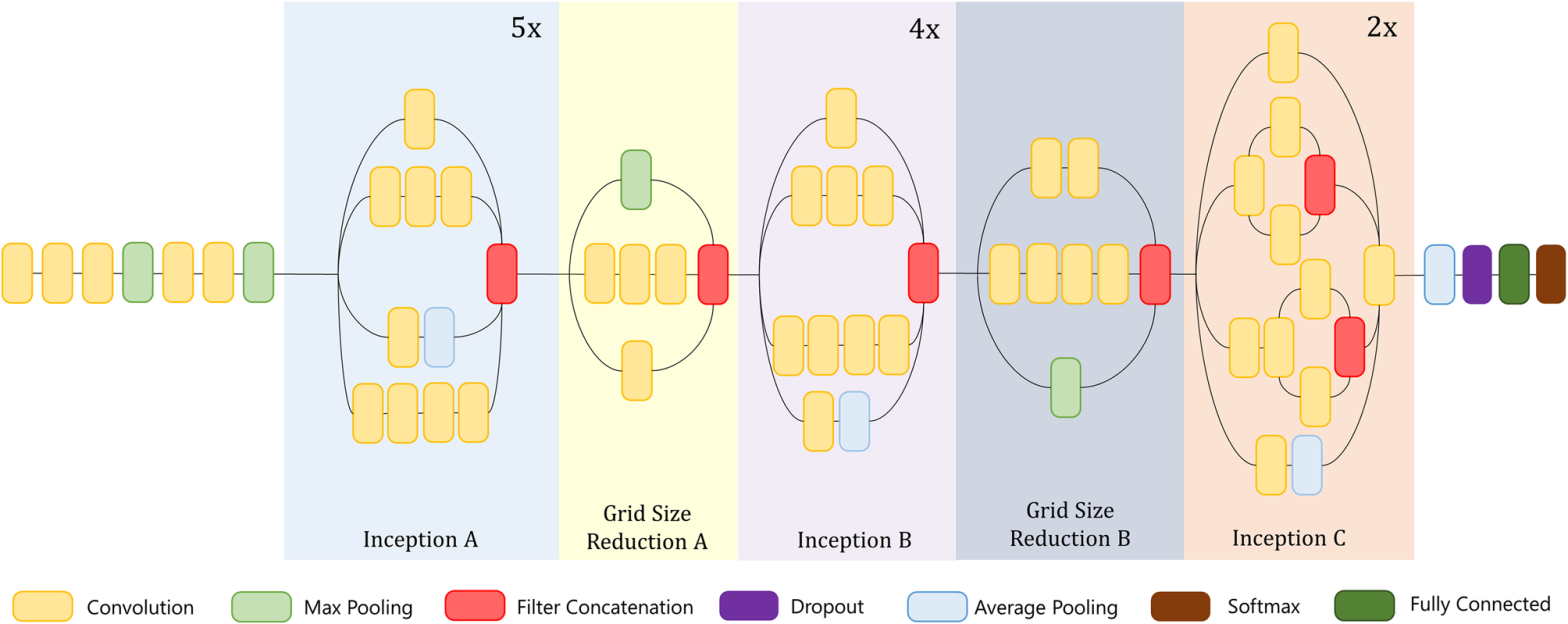
Architecture of the Inception V3 base model.

Inception v3^41^ is one of the most used deep learning models, belonging to the Inception family that uses various improvements like using an auxiliary classifier, factorized convolution operations, batch normalization, RMSProp optimizer, and label smoothing to mitigate the problems of the previous Inception models^46^. It takes an input image of size $299\times 299\times 3$ and produces feature maps of different dimensions in different layers. The inception block of Inception v3 allows us to utilize the facilities of using different filters of feature extraction from a single feature map. These features with different filters are concatenated and passed on to the next layer for deeper feature extraction. The architecture of the Inception v3 model used in the present work is shown in Fig. 8.

### Proposed fuzzy-ranking based ensemble using Gompertz function

The main motivation behind using a fuzzy rank-based approach is that, in such a technique, for every individual test case, priority is given to each classifier’s confidence in its predictions, unlike traditional ensemble approaches like the average rule, weighted average rule, etc., where classifiers need to be associated with a pre-defined fixed weight. We use the re-parameterized Gompertz function^49^ to generate the fuzzy ranks of each CNN classifier in detecting the COVID-19 cases from the CT-scans, and we fuse three CNN classifiers’ predictions, namely VGG-11, Wide ResNet-50-2, and Inception v3.

**Figure 9:**
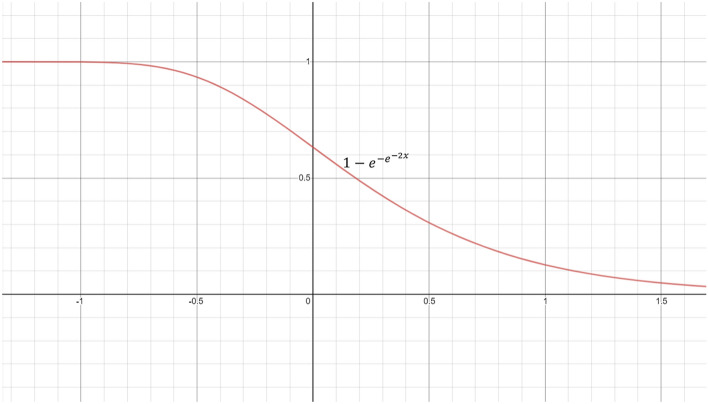
Displaying the re-parameterized Gompertz function used in the present study.

Biologically, the Gompertz model indicates an increase in mortality rate with increasing age, representing an increased vulnerability towards causes of death suffered by young adults. How rapidly this vulnerability enhances with age is depicted by the exponential term of the Gompertz function, where it is assumed that increasing age implies a greater probability of death^50^. Figure 9 shows the proposed re-parameterized Gompertz function, where the independent variable ‘*x*’ represents the predicted confidence score for a test sample by a classifier.

Let there be *M* number of decision scores (confidence factors of classifiers) $\{CF^{(1)}, CF^{(2)}, \ldots CF^{(M)}\}$ for each image ${\mathbf {I}}$. In our case, $M=3$, since we have used three CNN models to generate the confidence scores on the datasets. The decision scores are normalized which follow Eq. (1), where *C* is the number of classes in the dataset.

1 $$\begin{aligned} \sum _{c=1}^{C}CF_c^{(i)}=1.0; \text { }\forall i,\text { } i=1,2,3,\ldots ,M \end{aligned}$$

Corresponding to all samples belonging to different classes in the dataset, the confidence scores are used to generate the fuzzy ranks. The fuzzy rank for a class *c* using the $i^{th}$ classifier’s confidence scores is generated by the Gompertz function as in Eq. (2).

2 $$\begin{aligned} R_c^{(i)}=\left( 1-exp\left[ -exp\left[ -2.0\times CF_c^{(i)}\right] \right] \right) , \text { }\forall i,c;\;\; i=1,2,\ldots ,M; \text { } c=1,2,\ldots C \end{aligned}$$

The value of $R_c^{(i)}$ lies in the range [0.127, 0.632] where the smallest value 0.127 is analogous to rank 1 (best rank), i.e., a higher confidence gives a lower (better) value of rank. Now, if $K^{(i)}$ represents the top *k* ranks, i.e. ranks ${1,2,\ldots ,k}$, corresponding to class *c*, the fuzzy rank sum ($FRS_c$) and the complement of confidence factor sum ($CCFS_c$) are calculated as in Eqs. (3) and (4), respectively.

3 $$\begin{aligned}&FRS_c = \sum _{i=1}^{M} {\left\{ \begin{array}{ll} R_c^{(i)}, &{}\text {if } R_c^{(i)} \in K^{(i)} \\ P_{c}^{R}, &{}\text {otherwise } \end{array}\right. } \end{aligned}$$

4 $$\begin{aligned}&CCFS_c = \frac{1}{M}\sum _{i=1}^{M} {\left\{ \begin{array}{ll} CF_c^{(i)}, &{}\text {if } R_c^{(i)} \in K^{(i)} \\ P_{c}^{CF}, &{}\text {otherwise } \end{array}\right. } \end{aligned}$$

$P_{c}^{R}$ and $P_{c}^{CF}$ are the penalty values imposed on class *c*, if it does not belong to the top *k* class ranks. The value of $P_{c}^{R}$ is 0.632, which is calculated by putting $CF_c^{(i)} = 0$ in Eq. (2), and the value of $P_{c}^{CF}$ is set to 0.0. The penalty values ensure that class *c* does not become an unlikely winner.

The final decision score is realized by the product of $FRS_c$ and $CCFS_c$ which is used to generate the final predictions of the ensemble model. The final decision score (*FDS*) is calculated as in Eq. (5).

5 $$\begin{aligned} FDS_c = FRS_c \times CCFS_c \end{aligned}$$

The final predicted class of instance ${\mathbf {I}}$ of the dataset is calculated by finding the class having the minimum *FDS* value and is given in Eq. (6).

6 $$\begin{aligned} class({\mathbf {I}}) = \mathop {\arg \min }\limits_{{c=1,2,\ldots ,C}} \{FDS_c\} \end{aligned}$$

The computational complexity of the proposed ensemble approach is *O*(*n*) where ‘*n*’ is the number of classes in the dataset.

## Conclusion

With an increasing threat of novel coronavirus worldwide, early and accurate detection of COVID-19 becomes necessary because of the shortage of medical facilities faced by almost every country of the world. To this end, in this paper, we have proposed a fully automated COVID-19 detection framework employing deep learning that eliminates the need to undergo the tedious RT-PCR testing process but instead uses the more commonly available chest CT-scan images for classification. We have also demonstrated the application of fuzzy rank-based fusion on decision scores obtained from multiple CNN models to identify the COVID-19 cases. As far as our knowledge, the proposed framework is the first of its kind to form an ensemble model using the Gompertz function for COVID-19 detection. The low false-positive rate and high classification accuracies of 98.93% and 98.80% and sensitivities of 98.93% and 98.79% on the SARS-COV-2 and Harvard Dataverse datasets respectively, are the key achievements of the proposed method. The proposed framework has been compared to several techniques in literature, popular ensemble schemes adopted in different research problems and purely transfer learning-based approaches. In every case, the proposed fuzzy rank-based fusion scheme has outperformed the said methods, justifying its superiority.

In future, we aim to experiment with other CNN architectures as well as different fusion strategies to improve the performance. We also plan to validate the proposed method on other datasets, thereby proving the robustness of the proposed model. We may try to develop a more computationally efficient model for COVID-19 detection since an ensemble of different models requires a comparatively larger computation cost than a single model architecture. For this, we may try techniques like snapshot ensembling, etc. Also, from the loss curve analysis, we have seen that some models had overfitting issues, so we may try to address that using techniques like data augmentation, or we can try to acquire larger datasets to test on. We may also apply segmentation of the lung CT scans before classification to further enhance the recognition capability of the CNN models. We expect that the proposed model will be of great help to the medical practitioners for early detection which may lead to an immediate diagnosis of the COVID-19 patients since it can be used as a plug-and-play model where new test images can be passed through the saved model weights and the ensemble prediction can be computed.

## Data Availability

No datasets are generated during the current study. The datasets analyzed during this work are made publicly available in this published article.

## Code availability

The source codes used for the present research work are made publicly available in the GitHub repository: https://github.com/Rohit-Kundu/COVID-Detection-Gompertz-Function-Ensemble.
